# Beliefs about the factors that motivate prosocial sentiments among people in the privileged class of Pakistan during the COVID‐19 pandemic

**DOI:** 10.1111/josi.12506

**Published:** 2022-05-30

**Authors:** Ahsan Mehmood Ahmed, Shahid Rasool, Catherine Prentice, Muhammad Hissan Ahmad

**Affiliations:** ^1^ Capital University of Science and Technology Islamabad Pakistan; ^2^ Ghulam Ishaq Khan Institute of Engineering Sciences and Technology Topi, Swabi Pakistan; ^3^ Department of Marketing, Griffith Business School, Griffith Institute for Tourism Griffith University Australia; ^4^ University of Southern Queensland

## Abstract

Drawing upon the theories of empathy‐altruism and planned behavior, this study investigated beliefs about the factors that motivate prosocial sentiments among people in the privileged class of Pakistan during the COVID‐19 pandemic. In‐depth interviews were conducted with 31 participants who were deemed to be members of the privileged class within the class system of Pakistan. The results revealed nine themes including social interaction, peer influence, role models, collectivism, vicarious emotions, religiosity, capability, volition, and education.

## INTRODUCTION

Stimulating prosocial behavior around the globe is vital in times of a sustained pandemic such as the COVID‐19 pandemic. COVID‐19 has substantial social and economic impacts on countries such as Pakistan, India, Bangladesh, Sri Lanka, Nepal, Afghanistan, the Maldives, and Bhutan (Khan et al., 2020). For instance, in Pakistan a loss of US $2 billion was reported in labor force productivity, leading to job losses, and creating panic amongst the middle and lower‐middle class (Khan et al., 2020). The pandemic continues to have, a disastrous impact on global economies (Gates, 2020; Vetter et al., 2020; Xiang et al., 2020) affecting more than 50 million, and resulting in greater than 1.25 million deaths.

Many around the globe have exhibited prosocial behaviors to benefit others during the pandemic (Abel & Brown, 2020; Kawamura et al., 2021). Motivations for engaging in pro‐social behaviors are numerous. For example, Behler et al. (2020) found that an easy task motivated individuals towards prosocial behavior, whereas difficult tasks led to antisocial behavior. Karatas and Canli (2020) reported that God and religion were the most prevalent motivational reasons to donate money. Marshall et al. (2019) found that self‐compassion and empathy motivated prosocial behavior in adolescence. Developing positive and cooperative affiliation with children also motivates prosocial behavior (Torrens & Kartner, 2018). Motsenok and Ritov (2020) revealed that people with nominal finances compared to financially stable individuals were motivated towards prosocial behaviors.

Researchers often draw on empathy‐altruism theory (EAT) to understand the motivations behind prosocial behaviors (Batson et al., 2015). According to this theory, prosocial behaviors are evoked by feelings of empathy towards others. However, feelings may not be a sufficient antecedent for behavior. According to the theory of planned behavior (TPB) (Ajzen, 2011), behaviors generally result from attitudes, subjective norms, and perceived behavioral control. The two theories may function conjunctively and provide a more holistic perspective of individuals’ prosocial behaviors. Research to date has not attempted to integrate the two to understand the motives underlying these behaviors. The present study integrates the two theories to understand beliefs about the factors that motivate prosocial sentiments and, presumably prosocial behaviors, of people who were members of the privileged class in Pakistan during the COVID‐19 pandemic. The privileged class in Pakistan consists of wealthy segments of the society (Robison & Stubager, 2018) who have socio‐economic advantages with readily available financial and non‐financial resources (Albert el al., 2018). As members of the privileged class, they enjoy a variety of psychosocial, economic and social benefits.

The privileged are viewed as affluent. By occupation they fall into categories such as senior corporate executives, large business owners, politicians, film actors, sports celebrities, and feudal lords (Kotler & Armstrong, 2013). The privileged class is deemed to have the financial capacity to help oppressed and underprivileged groups within society (Gibson, 2014). They engage in prosocial behaviors manifested in volunteering, cooperating, donating, and complying with socially acceptable norms (Condon, 2019). Such engagement is particularly important during events such as pandemics, as it may alleviate some of the detrimental impacts on individuals and society more broadly. Nevertheless, pro‐social behaviors are voluntary and intrinsically driven and are not a legal requirement. Understanding the motives believed to underly prosocial sentiments may be conducive to eliciting prosocial behaviors to help address the negative impact of the ongoing COVID‐19 pandemic.

### Theories of empathy‐altruism (EAT) and planned behavior (TPB)

According to EAT, the arousal of feelings such as sympathy, compassion, and concern for others stimulates altruistic behavior, for instance, promoting welfare and charitable activities in society (Batson et al., 2015). The theory also posits that people exhibit altruistic behavior towards others through a helpful nature, acting morally, and placing the significance of helping others as an internal, rather than an external reward (Batson et al., 2015). Empathy‐altruism involves a focus on others’ interests rather than attention to self‐interests (Batson, 2014). Empathy has been associated with traits such as compassion, sympathy, and feelings of concern towards people in need (Stocks et al., 2009).

Whilst empathy and altruism are important to understanding prosocial behaviors, other factors highlighted by TPB are no less relevant. Social norms such as the influence of family members, peers, friends, and others within one's social circle, may stimulate individuals to exercise prosocial behavior within society (Trafimow, 2009). The TPB posits that behaviors or behavioral intentions arise from three antecedent conditions: attitudes, subjective norms, and perceived behavioral control. Although this conceptualization has been debated and criticized in the literature (see Gross et al., 2015), TPB has been applied to explain the relationships among perceptions, attitudes, and behaviors (Ajzen, 2011). Whilst acknowledging these antecedents play important roles in individuals’ behaviors, personal traits have long been recognized as valid behavioral predictors (Argyle & Little, 1972; Budaev & Brown, 2011). Among those traits, empathy and altruism have been extensively used to explain prosocial behaviors (Batson et al., 2015).

### Motivations for prosocial behavior

Many academicians have examined the factors that promote prosocial behavior. Some have focused on the role of environmental cues and social norms (e.g., Nook et al., 2016). Capraro et al. (2019), for example, suggested that emotional stimulation of norms—a moral nudge—motivated youth to make online charity donations, which had a long‐term influence on their personality. Gross et al. (2015) and Upshaw et al. (2015) established that parental socialization, such as the use of emotional words, self‐understanding, and instrumental helping, was a significant factor motivating children's prosociality.

Other work has focused on personality traits. Columbus (2021) found that personality traits such as honesty‐humility explain prosocial behaviors. Individuals with a more compassionate nature have also been shown to be more prone to engage in prosocial behavior (Yue & Yang, 2021). For example, Saetrevik (2021) indicated that a concern for collective societal actions during the pandemic shaped individuals’ prosocial behaviors. Gherghel et al. (2020) conducted a cross‐cultural study and found that traits in children, such as being agentic and displaying moral obligation, motivated them towards prosocial behavior. An online study conducted by Böckler et al. (2018) indicated that the traits of players in online gaming platforms, such as care and compassion, motivated prosocial behavior. Cotney and Banerjee (2019) and Davis et al. (2017) found that possessing high moral values motivated prosocial behavior in school students. Curry et al. (2018) and Gross et al. (2017) concluded that, concern for others boosted prosocial behavior in university students and motivated moral behavior in children (Dunfield et al., 2019), promoting helping, sharing, and acts of comfort. Schott et al.’s (2019) findings signified that interpersonal altruism motivated young police officers towards prosocial behavior. Nabila et al. (2021) found that, agreeableness was an individual trait that mediated the relationship between self‐compassion and prosocial behavior in vocational school students. Aziz and Abid (2018) found that social responsibility and altruistic personality motivated adults towards prosocial behavior.

Researchers have also emphasized the significance of investigating pro‐social behavior during the COVID‐19 pandemic. Behler et al. (2020) suggested that engaging undergraduate students in social work in the U.S motivated them to engage in prosocial behavior. Religious beliefs and the fear of God have also been associated with prosocial behavior among organizational employees in the U.S (Karatas & Canli, 2020). Motsenok and Ritov (2020) conducted an online study in 14 countries and found that financial security was associated with helping others among individuals 50 years and older. Shi et al.’s (2020) findings in China established that maternal and paternal attachment and peer attachment motivated children towards performing prosocial behavior. Similarly, Vishkin et al.’s (2020) research in Italy indicated that religiously inclined university students were more likely to display prosocial engagement.

A majority of studies on prosocial behavior have attempted to identify similar reasons for helping others, such as religiosity, empathy, compassion, and interpersonal altruism. These reasons primarily stem from EAT. However, other potential predictors of altruistic behavior highlighted within TPB may also help to explain the dimensions of prosocial behavior including sharing, helping and comforting. The purpose of the present study therefore was to integrate the two theories to explore the motives underlying the prosocial behaviors of the privileged class in Pakistan during the COVID‐19 pandemic.

## METHOD

### Participants

Interviews were conducted with 31 (28 men, three women) participants who resided in the province of Punjab, Pakistan, an affluent province that is home to more than 60% of the total population of Pakistan (Kugelman, 2013). Participants were between 30 and 40 years of age and Islamic. Twenty‐two were business owners and CEOs of multinationals or NGOs and all participants were considered members of the privileged class. That is, they had high social economic status and occupied the “best” occupations that were not necessarily earned (Hafeez, 1985). The privileged class also refers to people who are employed at the highest levels in the country, own businesses, are politicians, or are wealth heirs (Marshall, 1996). Members of the privileged class often receive special support and compensations. Participants must also have engaged in prosocial behaviors during the previous 5 years. Following Guetterman (2015), after 31 interviews, theoretical saturation was achieved and thus no additional participants were recruited.

### Interview questions

We developed interview questions, including how concerned are you about others during the COVID‐19 pandemic? What makes you feel concerned about others? Why are you concerned about others in the society? How motivated are you to help others in the society during the COVID‐19 pandemic? What motivates you to help others in the society? Why do you help others in the society? What two specific reasons motivate you to help others in the society? This procedure allowed the interviewer to probe and obtain unrestricted responses to establish motivational cues and common themes in line with empathy‐altruism and the theory of planned behavior (Straker et al., 2013). The purpose of the interview protocol was to ensure that all questions would be covered during interviews, while allowing probes and follow‐up questions (Furgerson et al. 2012; Turner, 2010). For example, during the interview on their beliefs about motivational cues, participants would be asked about their attitudes and the influence of subjective norms, which may have stimulated responses concerning their emotions towards helping others during the pandemic. In addition, participants would be asked to freely express their views on motivational cues which might affect their emotions and what motivational cues are triggered while helping others. Each interview lasted for approximately 20–25 min.

### Procedure

The researchers approached a few CEOs of big firms in Pakistan and explained the purpose of this research. Upon their agreement to interview, they were requested to introduce their associates who were in similar positions and social status to theirs. Hence, a snowball sampling technique was used to reach potential participants, enabling us to recruit hard‐to‐reach cases. This method has advantage of reaching the hidden suitable population for the purpose of this study. The interviews were conducted in English on Zoom in October 2020. The researchers conducted the open interview inquiry. Prior to the interview, consultancy was sought from several colleagues who had expertise in this area. Consistent with the approach suggested in Dworkin (2012), Creswell et al. (2006), and Dworkin (2012), the interviewers endeavored to gain unlimited responses and probe hidden motivations for their prosocial behavior. As a result, the findings were not limited to previously documented prosocial behavior conclusions. A single zoom interview session lasted from 40 to 55 min. The one‐to‐one zoom conversions were recorded for transcribing.

### Data analysis

We conducted a pilot study (Whittemore et al., 2001) in which 10% of the interviewees from the full sample were selected for review by external experts (Sim et al., 2018; Zohrabi, 2013). Two experts heading a welfare organization and holding 13 years of field experience in social work were approached to share their views on the interviews. The purpose of approaching these experts was to authenticate the validity of the pilot study results through the application of a peer examination procedure. Two experts having a PhD degree in marketing and more than 10 years’ experience of social work were asked to review the full interview responses for the prosocial interview questionnaire (Creswell et al., 2006). External validity depends on the underlying similarities between the Pakistani context and other global contexts (Zohrabi, 2013). However, after detailed examination, both experts agreed that the results derived from the pilot study participants could be generalized to a wider population in Pakistan (Sousa, 2014).

Interviews were transcribed verbatim, coded, using an interpretivist approach, for theme number, theme name, participant name, line number, and page number (Wargo et al., 2016). A total of 1008 units of text were coded into 87 raw themes. These 87 raw themes were further coded into 21 units of broader themes using the manual spreadsheet method (Wargo et al., 2016). Inter‐coder reliability of the content analysis of the open‐interview data was established through the use of coders (Kurasaki, 2000). Inter‐coder reliability measured using the Kappa coefficient was above .80 (McHugh, 2012). Broader themes from the coded information led to the revelation of latent themes through thematic development.

## RESULTS

**FIGURE 1:**
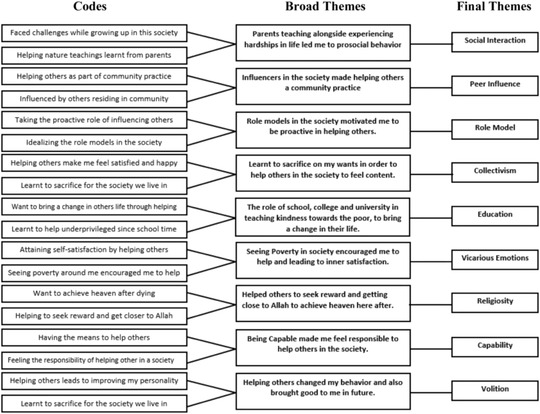
Thematic mapping for codes, broad themes, and latent themes

We identified nine broad themes in participants’ responses. We categorized five as external influences, namely, social interaction, peer influence, role model, collectivism, and education, and four as internal influences, namely, vicarious emotions, religiosity, capability, and volition. These themes are shown in Figure 1.

### External influences

#### Social interaction

Fifteen of the 31 participants came into interaction with individuals under perceived social pressures in their social circle who had faced challenges at different times between childhood and maturity. Also, as teenagers they had participated in different social activities leading to an understanding of social problems. Reflection on the life experiences of others within their social network motivated expressions of concern and the desire to help others in need. Participants shared that they were raised in an environment that taught them to help others. Interaction with people from different backgrounds was indicated as a major source for providing them with the motivation of helping people. They reported that parents played a significant role in providing an appropriate environment for building their children's social values, as shown in the following examples.

While growing up I came into interaction with people in the society belonging to multi ethnical back grounds (Hamza, age 31).

My parents raised me in an environment which taught me to look upon people who are financially weak (Talha, age 32).

I think it is what we absorb from our surroundings when we grow up (Zohaib, age 37).

Through working in an NGO [I] got a chance of interacting with people from different backgrounds (Abbas, age 37).

Learned through challenges while growing up in a society (Hasnain, age 39).

#### Peer influence

Not surprisingly, six of the 31 interviewees agreed that peers influenced their prosocial behavior during the COVID‐19 pandemic. Participants indicated that other members of the privileged class within their communities practiced the act of welfare. This belief reflected social norms that led participants to provide social assistance to poor people in the community. It was also suggested that family members and friends espoused the norms and values that encouraged the support of others, as shown in the following examples.

I enjoy the assistance of my community while doing welfare work (Talha, age 32).

Almost everyone in my culture and circle of influence reflects positive traits of prosocial behavior (Hissan, 39).

Basically, the culture around me of helping others influenced my social work behavior (Zainab, age 35).

### Role models

Eight of the 31 interviewees identified role models as a core motivation behind helping others during COVID‐19. Participants indicated that growing up in a society observing role models in the field of social work such as Imran Khan, Wasim Akram, Shahid Afridi, and Abdul Sattar Edhi influenced their own responses, as shown in the following examples.

I idolize Abdul Sattar Edhi as a community leader in social work (Hamza, age 31).

Amjad Saqib is a community leader in Pakistan, PM Imran Khan joined hands with him in building the community (Saad, age 34).

My cousin is my source of motivation for social work (Osama, age 30). I am motivated by the social work of Shahid Afridi (Musa, age 32).

#### Collectivism

Fifteen of the 31 interviewees were directed towards prioritizing collectivism as a motivational force to help others during the pandemic. The interviewees commented that living in a joint (or extended) family system as a child meant they learned the cultural and social values of their grandparents under perceived social pressure (TPB). Interviewees also indicated that the practice of living under one roof developed a behavior of sacrifice for other's needs, keeping aside one's own desires, and they felt that this sacrifice contributed to the betterment of society. For instance,

I have developed a sacrificing nature by living in joint family (Osama, age 34).

I learned social values by living in a combined family. This helped me to feel for others in the society (Hissan, age 39).

Living with my grandparents learned values for helping others (Musa, age 32).

Living in a family of 15 people taught me to think about others in the society and not be self‐centered (Zohaib, age 36).

#### Education

Eighteen of the 31 interviewees prioritized education as an external influence behind their prosocial behavior. They were of the view that their educational institutions played a pivotal role in controlling and directing their behavior (TPB) highlighting the significance of helping people in need in times of dire economic conditions. Several of the interviewees stated that prosocial behavior was imposed on them in the form of social work at school and at the college level, being required to undertake some community service. All interviewees commented that they had been taught from kindergarten to show concern for people in need in the society. Sympathetic characteristics and concern for others had been learned through social projects undertaken at school, college, and at the university level. For instance,

I have developed habit of helping others since school time (Azhar, age 36).

Prosocial behavior was imposed on me in college and [it] was compulsory to contribute certain hours [to] community service. My college grooming motivated me to help others (Saad, age 34).

I am proud of my school who taught me to help others in the society (Hissan, age 39).

Support of my teachers [has] encouraged me to help others in the community (Khalid, age 32).

### Internal influences

#### Vicarious emotions

Seventeen of the 31 interviewees emphasized vicarious emotions as a major reason for exhibiting prosocial behaviors. They were of the view that most people living in their vicinity did not have access to the basic necessities of life. For instance, women with infants begging, homeless elderly people sitting beside the road, and jobless daily wage earners asking for food. During the pandemic it was also necessary for communities to practice lockdowns for several days, daily wage earners with no food to feed their families. The interviewees stated that they found it difficult not to help people suffering from hunger and destitution, physical disability, financial crisis, and who may commit suicide, which aroused their feelings (EAT) and motivated them to help others. These acts of kindness brought inner contentment. For instance, the interviewees revealed:

I am very concerned, and I feel guilty for the poor people during this COVID‐19 (Saad, age 34).

I feel sorry for daily wage earners who have lost their jobs due to COVID‐ 19 (Shahab, age 39).

Being less privileged it is our responsibility to help them out (Talha, age 32).

As a nation we are below the poverty line and there are not many job opportunities (Moiz, age 39).

Basic medical facilities are not available to these poor people (Faizan, age 38).

It is not possible for me to turn a blind eye to the poverty, hunger and destitution, psychological traumas, financial crisis and suicidal tendencies (Masood, age 33).

Majority of the families were deprived of the daily income due to COVID‐ 19 (Abbas, age 37).

During lockdown, several people were jobless and had no source of income (Rida, age 34).

#### Religiosity

Twenty‐one of the 31 interviewees indicated religion as the core reason for engaging in prosocial behavior. Religious beliefs increased their level of concern for the poor. A few commented that their religious faith helped them to control and direct their behavior (TPB) and built their understanding of a need to show empathy for others. They illustrated that being Muslims by faith they had strong beliefs about achieving heaven in the hereafter. All interviewees mentioned that, the prime motive was heaven hereafter and doing good things would help to achieve this. It was also considered that humanitarianism during a global pandemic may bring them greater reward. For instance,

My religion has taught me to help others in the society (Azhar, age 33).

Our prime motive should be heaven and doing things which help me achieve heaven (Saad, age 34).

The purpose behind helping others is our religious beliefs (Shahab, age 39).

Our Islamic history has motivated us to help others (Osama, age 30).

God Almighty would bless me and I would be remembered by others (Zohaib, age 36).

It is our religious obligation to get involved in prosocial behavior (Moiz, age 39).

The other reason that motivated me was my religious believes (Yousaf, age 36).

I understand that humanity is the biggest religion, and Islam teaches us the lesson of humanity (Abbas, age 37).

#### Capability

Fourteen of the 31 interviewees stated that specific capabilities also influenced prosocial behavior. Growing up in a privileged family allowed financial resources to assist those in society living below the poverty line. Also, they stated that being rich and possessing unique skills aroused their feelings (EAT) and made them capable to serve humanity and also encouraged others to help people in need. They indicated that being capable and blessed with abundance of resources they felt it was their moral responsibility to undertake acts of sympathy towards the less fortunate residing in the community during the pandemic. For instance,

Being financially strong made me help others in the society (Shahab, age 39).

I am blessed and feel it is my responsibility to help others in the society (Osama, age 30).

Being a well settled businessman led me to prosocial behavior (Musa, age 32).

They are the ones who need money and help from people like us who belong to privileged family (Shanzeh, age 35).

#### Volition

Seven of the thirty‐one interviewees emphasized volition as the most important motivational cue. Their feelings were internally aroused (EAT) and believed that human character and personality was nourished by helping others. Also, they believed that, by spending one's wealth within the society brought future good. All the interviewees stated that, helping others enhanced their personality and brought benefit in the future. For instance,

The more you help others, the more you are investing and improving yourself (Saad, age 34).

If you are giving happiness to others it will come back to you too (Shahab, age 39).

Other reasons which motivated me to help others is character building that grooms one's personality. It removed any sense of arrogance that may exist in person and bring close to humbleness. So basically it [contributes] highly [to] personality and character building (Osama, 30).

## DISCUSSION

### External influences

#### Social interaction

Themes related to social interaction were mentioned 23 times by interviewees in 15 interviews. Inconsistent with previous research, which highlighted the influence of social support (Guo et al., 2017) using the empathy‐altruism hypothesis, the present research revealed irregular patterns. Findings suggest that the Pakistani privileged class acquired knowledge of welfare activities through social interaction, which led to prosocial behavior. We also found that being independent and liberal, the Pakistani privileged class invested the majority of their time in social interaction with peers, friends, and family, which influenced their sociological feelings and directed them towards providing financial assistance, food, and shelter to people in need (Vidal, 2008). The social interaction theme also highlights the significance of exploring the subjective norms for future prosocial behavior studies. In line with the findings Putra et al. (2021) and Rodrigues and Hewig (2021), the present study indicated that social interactions positively impacts and instills prosocial behavior in children and adults.

#### Peer influence

Peer influence was stated 15 times in six interviews. Consistent with previous research, it was found that the privileged class, through peer influence in their direct and indirect social groups, learned the act of human welfare. Notably, previous studies discussed peer influence in line with the theory of social learning (Van Hoorn et al., 2016). In comparison, the present research established the significance of peer influence in both empathy‐altruism and the theory of planned behavior. The study revealed that peer influence stimulated the concept of building moral values in the surrounding areas, collectively giving rise to an innovative social system prevailing on the principles of moral values. This finding is consistent with that of Joseph and Kuperminc (2021) and Osei (2021), indicating that peer influence plays a vital role observed prosocial behavior among individuals and groups.

#### Role models

Consistent with previous research on empathy‐altruism theory it was identified that, role models in society influenced people to exhibit prosocial behavior (Van Kleef & Lelieveld, 2022). Not surprisingly, this theme extended the empathy‐altruism theory in the theory of planned behavior (Hurd et al., 2011). It was observed that, these role models directly influenced the attitude of individuals towards helping the underprivileged. The finding also fits with the established theory of social learning, which indicates that positive role model behavior contributes towards attitudinal change in privileged class behavior (Van Hoorn et al., 2016). By modeling prosocial behavior, role models taught people in the privileged class how to socially interact with people in need. As they witness their role models being rewarded for their prosocial behavior, they may vicariously learn about the benefits associated with it. Exposure to role models’ prosocial behavior in the region of Punjab, Pakistan may help to build an on‐going welfare culture.

#### Collectivism

In comparison to the established literature based within individualist societies, in collectivist societies the decision to extend help to others is not a matter of individual decision making (Jassat et al., 2021). These decisions can be guided by a strong collectivist culture. In collectivism, individuals are expected to assist members within their social networks. It has been observed that, in comparison to individualist societies, collectivist societies tend to view extending social help towards others as a priority (Bahr et al., 2021). This research is also compatible with the theory of planned behavior. For example, individuals had developed an attitude to become engaged in prosocial behavior while residing in a collectivist culture. Perhaps more profoundly, individuals from collectivist societies might actually begin to care about the people whom they help. This may help to redefine group boundaries to include these individuals into their ingroups. It appeared that within a collectivist culture the threshold for becoming engaged in volunteering activities was raised, but once engaged in the experience, volunteering took on a life of its own.

#### Education

Education was referred to 22 times in 18 interviews. It was found that institutions such as family, school, college, university, and local communities played a significant role in developing an individual's social orientation towards prosocial behavior. It was stated that social work in schools created a link between individual prosocial behavior and the society at large. The majority of the interviewees commented that spending a couple of hours doing community work at the college level developed concern for their community. Others stated that community service modules should be introduced in the school curriculum at the national level, leading to develop a concern for the poor within the community. Social development was mentioned 41 times in 15 interviews.

### Internal influences

#### Vicarious emotions

Vicarious emotions were mentioned 41 times in 17 interviews. The study revealed that these members of the privileged class in an under‐developed economy displayed high concern for poverty. It is estimated that 22 million daily wage earners in the Punjab region were the core reason for motivating sympathetic emotions within the privileged class (Hassan & Farooq, 2015; Gilani, 2015; Sarwar & Abbasi, 2013). The study also found that the COVID‐19 pandemic led to complete lockdowns in the province of Punjab, leaving the majority of daily wage earners homeless and deprived of the basic necessities of life. This provided opportunities for the privileged class to display acts of compassion under these extreme circumstances.

#### Religiosity

Religiosity was mentioned 38 times in 21 interviews. Consistent with past studies, individuals practicing religious faith reported exhibiting traits such as being generous and were concerned about the poor people in society (La Ferle et al., 2021). It was also found that, facing under development and having limited employment opportunities at a national level poor people's major source of motivation was faith in God. This is thought to be because those below the poverty line had minimal attention from the government. The research showed that in the absence of government support for poor people, people in the privileged class saw a role to support society. Also, this role provided them a purpose in life through which they gained respect in the society (Guo et al., 2017).

#### Capability

Capability was cited 23 times in 14 interviews. As compared to previous literature it was found that members of the privileged class possessed several capabilities. This included wealth, knowledge, ambition, helpfulness, responsibility, honesty, and trustworthiness (Griffiths, 2017). Through utilizing these capabilities people in the privileged class enjoyed favorable reputations in society. Moreover, these capabilities helped them deliver prosocial behavior. Frequent social contact also allowed these traits to assist in forming a self‐identity amongst their social circles. These traits presented a feeling of uniqueness when compared to others in society. This distinction allowed them greater opportunity to focus on the social development of people in need.

#### Implications

This research explored and identified beliefs about the factors that motivate prosocial sentiments among people in the privileged class, highlighting social interaction, peer influence, role models, collectivism, vicarious emotions, religiosity, capability, volition, and education as motivators of prosocial behavior. The identification of nine latent motivations in times of crisis, such as the COVID‐19 sustained pandemic, provided an opportunity to extend and combine empathy‐altruism theory and the theory of planned behavior.

The emergence of new themes from previously unexplored dimensions provide a more meaningful and improved understanding of the factors that stimulate prosocial behavior among people in the privileged class in Punjab, Pakistan.

Previous work based on empathy‐altruism theory has highlighted the role of arousal feelings as antecedents of prosocial behavior (Schroeder & Graziano, 2015). The present qualitative endeavor suggested nine categories, including internal and external, that may activate prosocial behavior. For instance, external influences, including social interaction, peer influence, role models, and collectivism may lead to the extension of EAT and TPB in the field of social psychology. These cues may validate the relationship between individual mental states and socially fostered feelings, thoughts, and behaviors in relation to prosocial behavior. Similarly, internally motivated cues, including vicarious emotions, religiosity, capability, and volition, provide evidence that individuals differ based on psychological forces. Most consistently these cues validated that individuals’ psychological processes involve the construction of a coherent picture and its psychological processes in relation to the society. Consequently, we have developed a unique and dynamic set of characteristics. Moreover, the application of empathy‐altruism theory and the theory of planned behavior open new areas of interest for future prosocial behavioral studies. Theory building for prosocial behavior using attitude and subjective views may also reveal new ways to encourage support for others in society.

The present study also has implications for practitioners. Government‐affiliated intermediary organizations could embed these themes at a cultural level for positive societal outcomes. For instance, small and medium size enterprises (SMEs) could play a pivotal role in creating a link between society and recognized national brands for the development of a prosperous national culture. Secondly, role models could be promoted via the platforms of recognized global brands such as McDonalds, Nike, Coca Cola, and Pepsi. These brands could encourage the nation's youth to contribute towards society (Kivimaa, 2014). Likewise, government intermediaries could support educational institutions to develop a 6‐month social work module to help students understand the significance of a prosocial orientation towards society. The module could encourage students to learn skills such as self‐determination and cultural awareness, reveal inner strengths, and increase concerns related to social justice. This could be supported by field work in areas such as poverty relief fund raising, life skills education, community development social work, rural development, women's rights campaigns, child protection, and social health work (Martindale et al., 2017).

Prosocial behavior could also be incorporated into nationwide school programs. The education sector could create a broader culture in which students have the opportunity to see prosocial behaviors modelled by other students and adults. Module leaders could be trained to integrate value instruction into classroom management. Allowing students to participate in class decision making can promote an understanding of societal values, respect for others' opinions, and social responsibility. Cooperative behavior could be encouraged by assigning academic tasks in the classroom to pairs or small groups of students, which may help to promote students' ability to work together towards common goals. Adults within the school, including teachers, school administrators, cafeteria workers, and school bus drivers, could model caring and respectful behavior. Schools might also encourage prosocial behavior by using consistent positive disciplinary practices and extrinsic rewards to deter negative behavior, although these actions may not necessarily promote prosocial behavior. Praising accomplishments in specific domains and providing feedback may play a role in producing positive outcomes.

Individual sacrifice and concern for others may also lead to the art of sharing resources with poorer people in society. For instance, inspirational families living all over Pakistan could build prosocial networks of individuals residing in different geographic locations. These individuals could play a positive role in developing the attitudes of adolescents. Informal social gatherings organized by the youth could occur at the district and community level. The purpose of these gatherings would provide awareness to people about the significance of prosocial behavior in developing countries. SMEs across Pakistan could join as role models to expedite prosocial behavioral attitudes in the youth of Pakistan who represent 57% of the population (Ashraf et al. 2013).

### Limitations and future directions

This exploratory endeavor has limitations and provides insights for future research. The research was primarily directed towards highlighting the motivations of people in the privileged class towards prosocial behavior in times of crisis such as the COVID‐19 pandemic in the province of Punjab, Pakistan. A wider sample from other locations may provide greater insights. Evidence from past research indicates that the beliefs, morals, and social values of people in the privileged class towards people who are poor in times of crisis varies from one nation to another (Goodman, 2000). Consequently, a comparison of two or more nations would bring further insights to help understanding prosocial behaviors.

We also utilized open interviews, focusing on people within the privileged class. Quantitative research as well as comparisons of the prosocial behaviors of people in different classes would likely reveal additional insights. Comparing the proscocial behavior of people from different family backgrounds within the same social class may also enrich the findings. Finally, the motivations of people in the privileged class and those who are capitalists towards helping others may be different in times of crisis in Pakistan.

## CONCLUSION

In view of the dramatic impact of the COVID‐19 pandemic in Pakistan, prosocial behaviors from people in the privileged class were deemed to be important to rectify the negative influence on those less fortunate. The current study draws upon two theories (EAP and TPB) and in‐depth interviews with participants who were members of the privileged class in Punjab, Pakistan, to identify their beliefs about the factors that motivated them to engage in prosocial behavior. The findings of this study provide insightful information and new perspectives on motivation for prosocial behaviors.
